# Lithium Toxicity: A Case Report of Toxicity Resulting in a Third-degree Heart Block

**DOI:** 10.5811/cpcem.1442

**Published:** 2024-01-23

**Authors:** Samantha L. Gaetani, Alexandra M. Amaducci, Derek Fikse, Andrew L. Koons

**Affiliations:** * Lehigh Valley Health Network/University of South Florida Morsani College of Medicine, Department of Emergency and Hospital Medicine, Allentown, Pennsylvania; † Lehigh Valley Health Network/University of South Florida Morsani College of Medicine, Department of Emergency and Hospital Medicine, Division of Medical Toxicology, Allentown, Pennsylvania

**Keywords:** *case report*, *lithium toxicity*, *heart block*, *therapeutic index*, *hemodialysis*, *pacemaker replacement*

## Abstract

**Introduction:**

Lithium is a medication used to treat bipolar disorder. It has a narrow therapeutic index, which frequently causes toxicity in patients.

**Case Report:**

We present an unusual case of a 66-year-old female with a history of bipolar disorder on chronic lithium, who developed a third-degree heart block, encephalopathy, and acute renal failure because of lithium toxicity.

**Conclusion:**

This case highlights a rare but life-threatening case of complete heart block in the setting of lithium toxicity. The patient was treated with hemodialysis and pacemaker placement.

Population Health Research Capsule What do we already know about this clinical entity?

*Lithium can be beneficial as a mood stabilizer in patients with bipolar disorder, but its narrow therapeutic index frequently causes toxicity.*

What makes this presentation of disease reportable?

*Third-degree heart block as a result of lithium toxicity has only twice before been reported in the literature.*

What is the major learning point?

*Cardiotoxic effects of lithium toxicity are rare but can be life-threatening.*

How might this improve emergency medicine practice?

*Clinicians should be aware that lithium’s mechanism of action is incompletely understood and can present with a multitude of toxicities.*

## INTRODUCTION

Lithium has been approved by the United States Food and Drug Administration for treatment of bipolar 1 disorder since 1970.^1^ ^,^ ^2^ While its mechanism of action is incompletely understood, it is thought to act as a mood stabilizer by affecting neuronal plasticity.^1^ Lithium mimics sodium and uses the sodium channels in the body, specifically in the kidneys, to be transferred across cell membranes.^3^ It is also excreted similarly to sodium through the proximal tubules of the kidney.^3^ Lithium is rapidly absorbed in the gastrointestinal tract, reaching peak concentrations in 1–2 hours, and its half-life is 14–30 hours.^4^ The normal dose is 300–2,400 milligrams (mg) daily.^4^

While lithium can be beneficial as a mood stabilizer, its narrow therapeutic index frequently causes toxicity in patients.^5^ The therapeutic range for lithium is 0.6–1.2 milliequivalents per liter (mEq/L), and toxicity occurs when concentrations rise to 1.5 mEq/L or greater.^4^ The acute toxic dose of lithium is 1 mEq per kilogram (kg) or 40 mg/kg, which is about 20–30 tablets; acute on chronic and chronic toxicity can occur as well.^4^ Chronic toxicity results from drug interactions or changes to lithium excretion.^5^ For instance, dehydration, sodium depletion, or excessive sodium resorption can lead to chronic lithium toxicity.^5^ Third-degree heart block as a result of lithium toxicity has only twice before been described in the literature.^6^ ^,^ ^7^

## CASE REPORT

A 66-year-old female with a past medical history of hypothyroidism, type 2 diabetes, hypertension, and bipolar disorder presented to the emergency department (ED) for fatigue, depression, and tremors. Most of the history was provided by the patient’s son, who estimated that the symptoms had been occurring for 3–7 days. The patient also reported decreased appetite and decreased fluid intake. She reported taking her medications as prescribed. Her home medications included lithium 300 mg twice daily, levothyroxine 88 micrograms daily, bisoprolol-hydrochlorothiazide 10–6.25 mg daily, and atorvastatin 80 mg daily. The patient denied any abdominal pain, nausea, vomiting, dizziness, dysuria, fever, headache, syncope, or shortness of breath.

On initial presentation, the patient had a heart rate of 34 beats per minute, blood pressure 157/59 millimeters of mercury, oxygen saturation of 100% on room air, respiratory rate of 14 breaths per minute, and a temperature of 97.5° Fahrenheit. On her initial physical exam pertinent findings included somnolence, although she answered questions appropriately, mild tremors of the upper and lower extremities, and marked bradycardia.

**Image 1.**
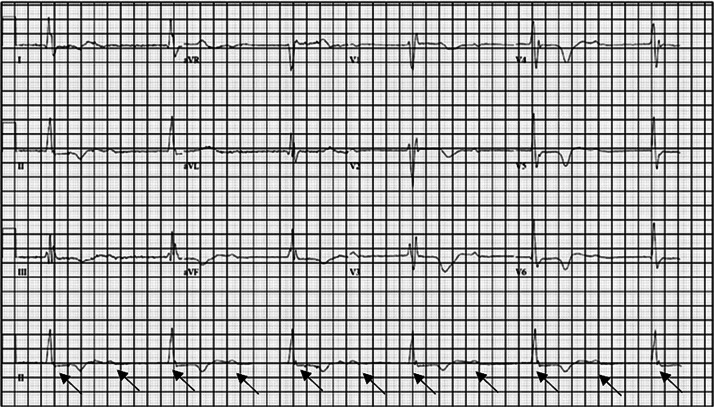
This electrocardiogram shows third-degree heart block at a rate of 33 beats per minute. There is QRS complex widening at 174 milliseconds (ms) with Right Bundle Branch Block and a prolonged QTc interval at 560 ms. (Normal QRS is 70–100 ms and normal QTc less than 450 ms for males and less than 470 ms for females). The p-waves (indicated by black arrows) do not line up with the QRS complexes, thus demonstrating a third-degree heart block.

An electrocardiogram was completed (Image 1) and demonstrated a third-degree heart block. A lithium concentration of greater than 3 mEq/L (reference range: 0.60–1.2 mEq/L, with 3 mEq/L the maximum detectable in our laboratory) was detected. A send-out test determined her initial lithium concentration to be 3.8 mEq/L (0.5–1.2 mEq/L). Other pertinent abnormal labs included sodium 130 mEq/L (135–145 mEq/L), creatinine 1.89 mg per deciliter (dL) (0.40–1.10 mg/dL), and glomerular filtration rate 27 (reference range: >60). Given the bradycardia with prolonged QRS complexes and QTc intervals with lithium toxicity, our bedside medical toxicology consultation service recommended normal saline, two ampules of sodium bicarbonate, and two grams of magnesium sulfate. However, the patient developed worsening encephalopathy, and hemodialysis (HD) was recommended. She was taken to the catheterization lab to have a temporary transvenous pacemaker placed.

**Image 2.**
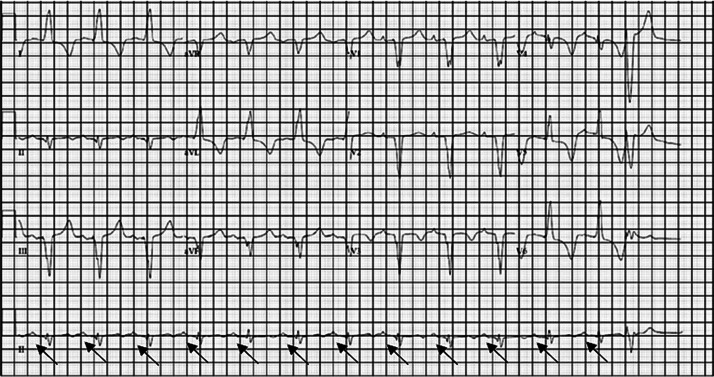
Electrocardiogram after a permanent pacemaker was placed. Atrial-sensed ventricular-paced rhythm with occasional premature ventricular complexes at a rate of 82 beats per minute. There is a p-wave (black arrows) before every QRS complex with regular intervals, indicating the patient is no longer in third-degree heart block.

Throughout her intensive care unit (ICU) stay, the patient underwent emergent dialysis, which resulted in improvement of her mental status and laboratory abnormalities. The third-degree heart block, however, had not resolved. A permanent Medtronic DC pacemaker was subsequently placed (Image 2).

## DISCUSSION

Lithium is a xenobiotic that can cause severe toxicity when it exceeds therapeutic range.^5^ The lithium concentration in our patient was 3.8 mEq/L. While she had been on lithium for many years, over the prior several days she had decreased oral intake. This likely led to dehydration and renal dysfunction. In addition, this patient was on hydrochlorothiazide, potentially contributing to reduced renal elimination and dehydration with subsequent lithium accumulation. Symptoms of toxicity occur on a spectrum. Mild toxicity may manifest as nausea, vomiting, tremors, drowsiness, hyperreflexia, hypertonia, fasciculations, slurred speech, ataxia, and apathy, while severely poisoned patients may have coma, seizures, hyperthermia, delirium, and death.^4^

Lithium can also cause nephrogenic diabetes insipidus, hyperparathyroidism, hypothyroidism, and rarely hyperthyroidism.^4^ Our patient was initially somnolent and had tremors consistent with mild symptoms; however, she became encephalopathic and hemodynamically unstable leading to the decision to start HD. Hemodialysis is the mainstay of treatment for severe lithium toxicity in patients with seizures, a severely abnormal mental status, hemodynamic instability, or in a patient who is unable to excrete lithium through the kidneys.^4^ ^,^ ^8^

This case is unusual due to the associated third-degree heart block. Lithium toxicity infrequently causes cardiotoxicity.^9^ There have been reports of cardiac arrythmias, t-wave changes, and even an unmasking of Brugada syndrome.^9^ T-wave flattening/inversions and depressed ST segments can commonly be seen with lithium toxicity; however, bradycardia, sinus node arrest, third-degree heart block, and unmasking of Brugada morphology have rarely been reported.^4^ ^,^ ^9^ These cardiotoxic effects are believed to be caused by lithium interacting with sodium channels in the cardiac tissue resulting in dysfunction in the cardiac membrane physiology.^9^ Similar to the neurologic manifestation of lithium poisoning termed SILENT (Syndrome of Irreversible Lithium-Effectuated NeuroToxicity), it does not immediately or potentially ever resolve with removal of lithium.^10^ It may be reasonable to hypothesize that the same cardiotoxic effects are caused and persist despite removal of lithium, although the mechanism is not known.

## CONCLUSION

While lithium is a medication that has been used for more than 50 years, its mechanism of action is still incompletely understood, and it can present with a multitude of toxicities. This case highlights a rare but life-threatening case of complete heart block in the setting of lithium toxicity.
